# Combined sustained release of BMP2 and MMP10 accelerates bone formation and mineralization of calvaria critical size defect in mice

**DOI:** 10.1080/10717544.2018.1446473

**Published:** 2018-03-08

**Authors:** Ricardo Reyes, Jose Antonio Rodríguez, Josune Orbe, María Rosa Arnau, Carmen Évora, Araceli Delgado

**Affiliations:** a Department of Biochemistry, Microbiology, Cell Biology and Genetics, Universidad de La Laguna, La Laguna, Spain;; b Institute of Biomedical Technologies (ITB), Center for Biomedical Research of the Canary Islands (CIBICAN), Universidad de La Laguna, La Laguna, Spain;; c Laboratorio de Aterotrombosis, Área de Ciencias Cardiovasculares, CIMA-Universidad de Navarra, Pamplona, Spain;; d CIBER de Enfermedades Cardiovasculares (CIBER-CV), Madrid, Spain;; e IdiSNA-Health Research Institute of Navarra, Pamplona, Spain;; f Servicio de Estabulario, Universidad de La Laguna, La Laguna, Spain;; g Department of Chemical Engineering and Pharmaceutical Technology, Universidad de La Laguna, La Laguna, Spain

**Keywords:** BMP-2, MMP10, sustained release, bone repair, mineralization, histomorphometry

## Abstract

The effect of dual delivery of bone morphogenetic protein-2 (BMP-2) and matrix metalloproteinase 10 (MMP10) on bone regeneration was investigated in a murine model of calvarial critical-size defect, hypothesizing that it would result in an enhanced bone formation. Critical-size calvarial defects (4 mm diameter) were created in mice and PLGA microspheres preloaded with either BMP-2, MMP10 or a microsphere combination of both were transplanted into defect sites at different doses. Empty microspheres were used as the negative control. Encapsulation efficiency was assessed and *in vivo* release kinetics of BMP-2 and MMP10 were examined over 14 days. Histological analyses were used to analyze bone formation after four and eight weeks. Combination with MMP10 (30 ng) significantly enhanced BMP-2 (600 ng)-mediated osteogenesis, as confirmed by the increase in percentage of bone fill (*p* < .05) at four weeks. Moreover, it also increased mineral apposition rate (*p* < .05), measured by double labeling with tetracycline and calceine. MMP10 accelerates bone repair by enhancing BMP-2-promoted bone healing and improving the mineralization rate. In conclusion combination of MMP10 and BMP-2 may become a promising strategy for repair and regeneration of bone defects.

## Introduction

Fractures are one of the most frequent injuries of the musculoskeletal system. Optimal treatment of fracture requires a complete knowledge of the complex process of bone repair and regeneration, critical for the maintenance of mobility and structural integrity.

Growth factors (GFs) of TGFβ family, including bone morphogenetic proteins (BMPs), play important roles in skeletal development (Chen et al., 2012). BMPs signaling leads to the expression and activity of genes necessary for osteoblast differentiation (Song et al., 2009). Particularly, BMP-2, one of the most important cytokines regulating osteoblasts differentiation, plays relevant roles in a variety of cellular functions ranging from embryogenesis, cell growth, and differentiation to bone development and the repair of bone fractures (Rosen, 2009; Dimitriou et al., 2011).

Matrix metalloproteinases (MMPs) are the members of a family of calcium-dependent zinc-containing proteinases, which are able to cleave a wide range of extracellular proteins, including several components of the extracellular matrix (ECM). MMPs have pivotal functions in physiology and pathophysiology, playing important roles in cancer but also in normal development, wound healing and tissue remodeling after damage. Several MMPs have been involved in bone homeostasis and fracture healing, such as MMP2 (Lieu et al., 2011), MMP9 (Colnot et al., 2003) and MMP13 (Stickens et al., 2004; Kosaki et al., 2007). Among them, MMP10 (stromelysin-2) has been involved in bone growth (Ortega et al., 2004), cartilage degradation (Barksby et al., 2006) and wound healing (Gill & Parks, 2008). MMP10 can be induced by inflammation (Montero et al., 2006; Orbe et al., 2009), and its increase in vascular pathologies (Rodriguez et al., 2008) has been associated with atherosclerosis (Coll et al., 2010; Martínez-Aguilar et al., 2015) and thrombin generation (Orbe et al., 2009). Besides, a profibrinolytic function has been reported for active MMP10, by enhancing tissue plasminogen activator (tPA)-dependent fibrinolysis (Orbe et al., 2011). MMP10 has been shown to be involved in tissue repair processes using different experimental models of damage, such as hind limb ischemia (Gómez-Rodríguez et al., 2015), skeletal muscle (Bobadilla et al., 2014) and liver injury (García-Irigoyen et al., 2014).

Interestingly, MMP10 has been shown to be expressed in osteoblasts at sites of bone formation as well as in chondrocytes of the growth plate in neonatal ribs in human (Bord et al., 1998). Moreover, MMP10 augmented the differentiation of myoblastic cells into osteoblastic cells induced by BMP-2, but not in the absence of exogenous BMPs, leading to the proposal that MMP10 promotes the differentiation of myoblasts into osteoblasts by interacting with the BMP signaling pathway (Mao et al., 2013).

Nowadays, it is well known that GFs and active substances must act in a pre-established order and during a specific period of time to form functional tissue. Hence, the important role played by the controlled release systems of these functional molecules involved in bone regeneration (De la Riva et al., 2010; Lee et al., 2011; Rodríguez-Évora et al., 2013; Del Rosario et al., 2015a,b). The repair of critical bone defects remains a major clinical orthopedic challenge. Therefore, according to the involvement of MMP10 in the BMP signaling pathway previously reported *in vitro*, we hypothesized that bone regeneration could be improved *in vivo* by combining both molecules. In the present study, using a controlled release system, we investigated whether MMP10 can enhance BMP-2-promoted bone repair *in vivo*, in a murine model of critical-size bone defect. Time residence in the defect site of free and pre-encapsulated MMP10 and BMP-2 was assayed. Afterwards, a microsphere suspension containing BMP-2 or MMP10 was proposed as scaffold to test our hypothesis.

## Materials and methods

The poly (lactic-co-glycolic acid) (PLGA) used was Resomer^®^ RG504 (Evonik, Darmstadt, Germany). Pluronic F127^®^ was purchased from (Sigma-Aldrich, St Louis, MO, USA). The BMP-2 (GenScript, Piscataway, USA, lot:P50011308) showed an ED_50_ = 0.86 µg/mL measured by its ability to induce alkaline phosphatase production by C2C12 cells.

### Expression and purification of recombinant human proMMP10

Human proMMP10 was produced as previously described (Orbe et al., 2011). Briefly, full-length human proMMP10 was inserted into pcDNA 3.1-V5-His (Invitrogen, Thermo Fisher Scientific, Carlsbad, CA, USA) expression vector. HEK293 (Sigma) cells were transfected with Lipofectamine 2000 (Invitrogen, Thermo Fisher Scientific) and grown before clonal selection in the presence of geneticin (Calbiochem, Darmstadt, Germany). Supernatants were screened for the production of proMMP10 by ELISA (R&D Systems, Abingdon, UK) and Western blot with an anti-MMP10 antibody directed to the catalytic domain of the protein (MAB9101, R&D Systems). Cells were grown in Hyperflasks (Corning, Lowell, MA, USA), switched to serum free DMEM (Gibco, Thermo Fisher Scientific, Paisley, UK) and supernatants were collected every 48 h, concentrated (VivaFlow 200; 30 kDa cut off, Sartorious, Goettingen, Germany) and stored at −20 °C until purification. A two-step purification strategy was designed, comprising immobilized metal affinity chromatography on a cobalt charged resin (Co-MAC, Novagen, Merck, San Diego, CA, USA), followed by immunoaffinity chromatography with a HiTrap NHS-activated HP column (GE Healthcare, Uppsala, Sweden) coupled with an anti-MMP10 antibody. ProMMP10 was dialyzed against TN buffer (50 mM Tris-HCl pH 7.5, 150 mM NaCl), sterile filtered and stored at −80 °C. Sample purity was assessed by sodium dodecyl sulfate polyacrylamide gel electrophoresis (SDS-PAGE) followed by staining with Gelcode Blue Stain Reagent (Thermo Fisher Scientific).

### Animal experiments

Seventy one male CD-1 mice weighing approximately 30 g, supplied by the animal house of the University of La Laguna, were used in this study. All animal experiments were carried out in conformity with the European Directive (2010/63/UE) on Care and Use of Animals in Experimental Procedures. Furthermore, the animal protocols were approved by the Ethics Committee for Animal Care of the University of La Laguna. The animals, before and after the surgery, were housed under a light/dark cycle of 12 h, at 22 °C and with food and water *ad libitum*. Surgery was made under aseptic conditions.

### Surgical procedure

Surgery was carried out under general anesthesia with isoflurane and the animal body temperature was kept at 37 °C with a heated platform. Briefly, the calvaria bone was exposed and a 4 mm circular area was delimited with a biopsy punch. Then, a 4 mm circular transosseous defect was made with a trephine bur (Rodríguez-Évora et al., 2013). The delivery system was inserted in the defect and the skin was stapled. After recovery from surgery, animals were allowed free movement, food and water uptake. Analgesia consisted in buprenorphine administered subcutaneously (0.05 mg/kg) before surgery and paracetamol (70 mg/100 mL) in water, during three days post-surgery.

### Delivery system preparation

Microspheres of BMP-2 and MMP10 were prepared by a double emulsion (w/o/w) method. Briefly, the aqueous phase of BMP-2 microsphere consisted of 100 µL of a 0.5 µg/µL, 0.25 µg/µL or 0.125 µg/µL BMP-2 in polyvinyl alcohol (PVA) solution (0.1%), depending on the dose (Table 1). The aqueous phase for MMP10 microspheres was 100 µL of a 25 ng/µL or 2.5 ng/µL MMP10 in PVA solution (0.1%), depending on the dose to be administered (Table 1). To prepare the first emulsion, the protein solutions were vortexed with 1 mL of a PLGA methylene chloride solution (50 mg/mL) for 3 min. The formed emulsion was poured into 50 mL of a 0.5% PVA solution and stirred for 1 minute at 1000 rpm (Silverson Homogenizer L4RT, Chesham, UK). Then, to evaporate the organic solvent, the suspension was maintained under magnetic agitation for 1 h. Microspheres were collected by filtration and freeze-dried.

**Table 1.** Experimental groups (*n* = 8 mice) for bone repair and mineralization rate evaluation.

Group	Treatment
Control (C)	Empty defect of 4 mm of diameter
Blank	Suspension of 2 mg of blank microspheres
BMP-100	100 ng BMP-2 in 2 mg of microspheres suspension
BMP-300	300 ng BMP-2 in 2 mg of microspheres suspension
BMP-600	600 ng BMP-2 in 2 mg of microspheres suspension
BMP-600-MMP3	600 ng BMP-2 and 3 ng MMP10 in 2 mg of microspheres suspension. Ratio 200:1
BMP-600-MMP30	600 ng BMP-2 and 30 ng MMP10 in 2 mg of microspheres suspension. Ratio 20:1

The implanted system consisted in a suspension of 1 mg of BMP-2 or MMP10 microspheres completed to 2 mg with blank microspheres or combination of 1 mg of microspheres loaded with BMP-2 and 1 mg of microspheres loaded with MMP10, depending on the experimental group (Table 1), in 7 µL of a 15% aqueous solution of Pluronic F-127^®^. Microspheres were morphologically characterized by scanning electron microscopy (SEM, Jeol JSM-6300, Tokyo, Japan) and sized using laser diffractometry (Mastersizer 2000, Malvern Instruments, Malvern, UK).

BMP-2 and MMP10 were labeled with ^125^I according to the iodogen method (Fraker & Speck, 1978; Del Rosario et al., 2015a) to determine encapsulation efficiency and release assays. Labeling yield and radiolabeling stability of ^125^I-proteins were checked by thin layer chromatography as previously described (De la Riva et al., 2009).

Encapsulation efficiency was determined by measuring the radioactivity of three aliquots of 3 mg of each lot of ^125^I-BMP-2 (M-^125^I-BMP) or ^125^I -MMP10 (M-^125^I-MMP) loaded microspheres in the gamma counter (Cobra II, Packard, Downers Grove, IL, USA).

### *In vivo* release assays

*In vivo* protein release assays were carried out with a non-invasive method as previously described and validated (Delgado et al., 2006). Briefly, a probe-type gamma counter (Captus, Capintec Inc., Ramsey, NJ, USA) was placed onto the defect site of groups of 5 sedated mice each and the remaining radioactivity was measured at 27 keV. The initial measure (time = 0) was considered the given dose (100%).

The *in vivo* BMP-2 release assay was carried out in one group of mice implanted with the M-^125^I-BMP-2 system. In addition, to check the effect of the microencapsulation on the MMP10 release profile, assays were carried out in a group implanted with free ^125^I-MMP10 (S-^125^I-MMP) and another group implanted with the M-^125^I-MMP microspheres suspension. The system with S-^125^I-MMP was prepared with 2 mg of blank microspheres and the MMP10 solution incorporated in the Pluronic F-127^®^ solution. As the polymer/protein ratio in the microspheres was high enough to assume no effect on the release rate, the *in vivo* release assays were made only with the high doses of protein.

### Histology and histomorphometrical evaluation

Defects of 56 mice divided in seven groups of eight mice each (four animals per time point) were examined to determine the bone-regenerative effect of the combination of BMP-2 and MMP10 at four and eight weeks postimplantation (Table 1). To label the mineralization front, the animals were injected with oxytetracycline-HCl (40 mg/kg, IM) and calcein blue (15 mg/kg, SC), 12 and four days before euthanasia, respectively.

After tissue fixation in 10% formalin solution (pH 7.4), undecalcified bone specimens were prepared for histological analysis as previously described (Rodríguez-Évora et al., 2013). Sections were stained with Goldner's Trichrome to identify new bone formation, or left unstained for detection of fluorochrome labels, and analyzed by light microscopy (LEICA DM 4000B, Barcelona, Spain).

For histomorphometrical analysis, all sections per specimen were evaluated using computer based image analysis software (Leica Q-win V3 Pro-image analysis system, Barcelona, Spain). We defined a region of interest (ROI) consisting of a circular area of 12.5 mm^2^, the center of which coincided with that of the defect site. This region covered the entire defect surface and was limited by the host bone. Within this ROI, newly formed bone was distinguished from scaffold material through structure and color differences. New bone formation was expressed as a percentage of repair in relation to the total area of the defect. The distance between tetracycline (green) and calcein blue labels was measured under ultraviolet light for calculation of mineral appositional rate (MAR). Given the characteristics of the bone growth, and therefore the irregularity of the mineralization fronts, 10 measurements at random points were made between both labeling fronts in 10 different sections along the defect in all the animals of each experimental group. The average was divided by the time elapsed between the administration of each fluorochromes, and this value was represented as the MAR.

Statistical analysis was performed with SPSS.21 software. We have compared the different treatments at each time point (four weeks and eight weeks) by means of a one-way analysis of variance (ANOVA) with a Tukey multiple comparison post-test. Significance was set at *p <* .05. Results are expressed as means ± SD.

## Results

### System characteristics and *in vivo* release

The mean volume diameter of the BMP-2 microspheres was 67 µm, 80% of the particles were in the range of 43–89 µm and the encapsulation efficiency was 65.5% ± 6.8%. MMP10 microspheres were similar in terms of size (52 µm mean diameter, 80% in the 24–80 µm range) and encapsulation efficiency (67% ± 4.9%).

**Figure 1.**
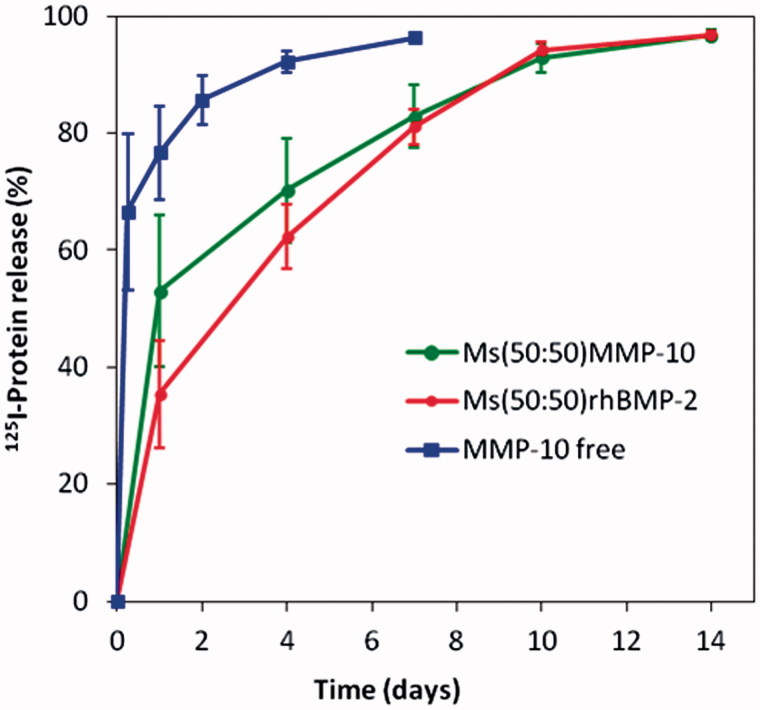
^125^I-BMP-2 and ^125^I-MMP10 (%) remaining at the implantation site after injection of free^125^I-MMP formulation (S-^125^I-MMP) and after injection of the microsphere systems M-^125^I-MMP and M-^125^I-BMP into the calvaria defects in mice.

*In vivo*, the remaining protein at the defect site was measured in the groups S-^125^I-MMP for one week and for two weeks in M-^125^I-MMP and M-^125^I-BMP (Figure 1). The S-^125^I-MMP curve represents the elimination kinetics of MMP10 from the bolus-treated calvarial defects. As expected, the release rate was faster when the ^125^I-MMP was free in the system S-^125^I-MMP: 80% of the protein was cleared from the implantation site within the first day and more than 90% in one week. By contrast, when proteins were encapsulated in the microspheres, 50% was released from M-^125^I-MMP and 35% from the M-^125^I-BMP within the first day, while two weeks were required to deliver the entire dose. After the initial burst effect observed with M-^125^I-MMP, higher than that of M-^125^I-BMP, the release rate during the period from the first to the 10th day was slower, 4.4% of MMP10/day against 6.1% of BMP-2/day.

According to the release profiles of both proteins (Figure 1), the BMP-2: MMP10 ratio in the groups implanted with the combination of BMP-2 microspheres with MMP10 microspheres was kept in the range of 100 % ± 50% of the initial 20:1 or 200:1 throughout the release period.

### Histological and histomorphometrical evaluation

First, the three doses of BMP-2 were assessed to choose the best one to be combined with MMP10. Animals underwent X-ray imaging four weeks after the procedure and radiological images showed a slightly better response in the groups treated with 600 ng BMP-2 than in the lower doses’ groups. Therefore, the 600 ng dose of BMP-2 in microspheres system was chosen to be combined with 3 and 30 ng of MMP10 in microspheres, to keep the 200:1 and 20:1 ratios.

**Figure 2.**
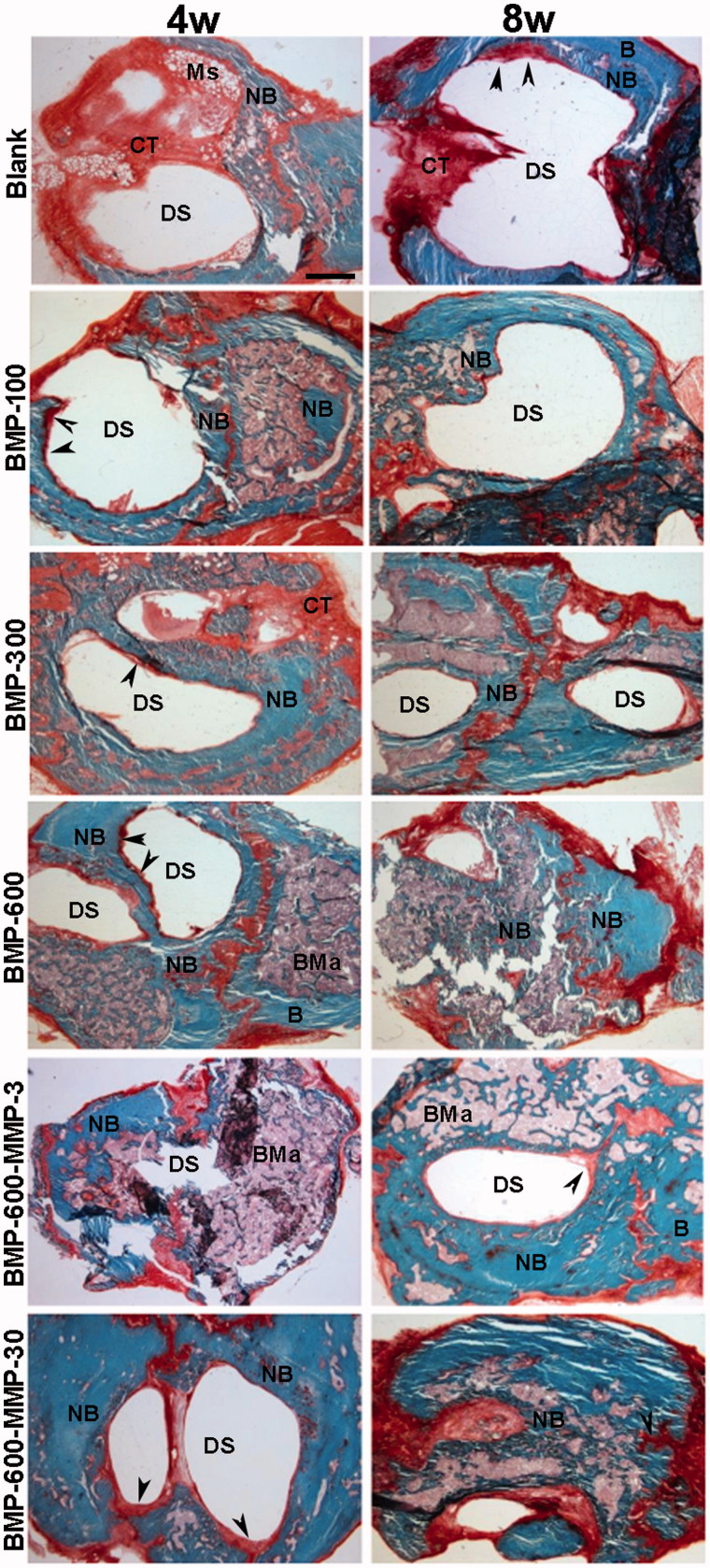
Representative images of the defect in the different experimental groups at 4 and 8 weeks post-implantation. Photomicrographs of horizontal sections of the calvarial defects implanted with systems loaded with 100, 300 and 600 ng of BMP-2 in microsphere and the systems containing a combination of BMP-2 and MMP-10 in microspheres: 600 ng of BMP-2:3 ng of MMP10 (ratio 200:1) and 600 ng of BMP-2: 30 ng of MMP10 (ratio 20:1). Arrowheads in the different images indicate active areas of osteosynthesis in the defect margins. B: host bone; BMa: bone marrow; CT: connective tissue; DS defect site; NB: newly formed bone; Ms: microspheres. Scale bar 1 mm.

**Figure 3.**
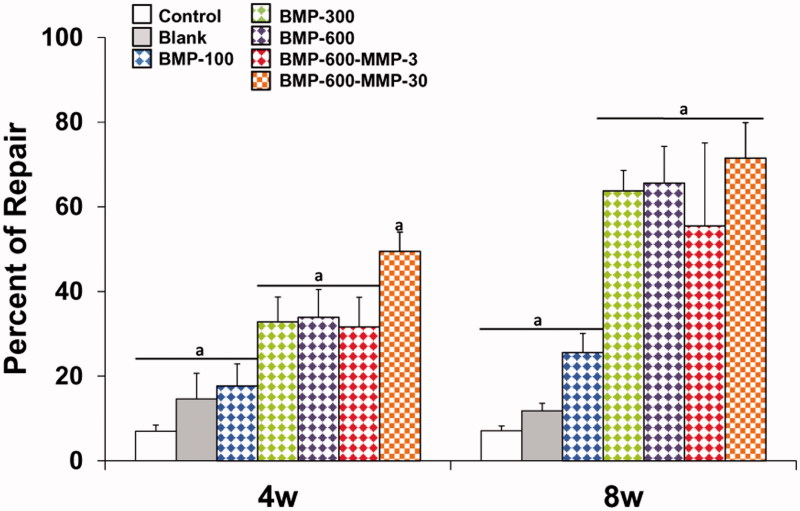
Histomorphometrical analysis. Comparison of the percentages of repair (%) among the different experimental groups at different experimental time points. Data presented as means ± SD. The identical letter on different bars indicates significant differences (*n* = 4), *p* < .05.

Histological and histomorphometric analyses showed no repair in both control (empty defect, data not shown) and blank groups (Figures 2 and 3), throughout the experimental period. Microspheres immersed in connective tissue could be observed four weeks post-implantation only in the blank group (Figure 2). The BMP-300 and BMP-600 groups showed similar percentages of repair, lower than 40% at 4 weeks and significantly higher than the blank and BMP-100 groups (Figures 2 and 3), in which the defect was observed almost intact, partially filled with connective tissue (Figure 2). In the group treated with the combination of BMP-2 and the low dose of MMP10, ratio 200:1, the response was similar to the BMP-300 and BMP-600 groups (Figures 2 and 3). However, the combination with high dose of MMP10, BMP-600-MMP30, ratio 20:1, showed a reparative response significantly higher than the other experimental groups, nearly 50% (Figures 2 and 3).

The results 8 weeks post-implantation showed a significant increase in the repair response in all treated groups as compared to 4 weeks, except for the BMP-100 group (Figures 2 and 3). The percentages of repair ranged from 55%, observed in the BMP-600-MMP3 group, and 72% in the BMP-600-MMP30 group, with no statistically significant differences among them (Figure 3). The regeneration process was clearly observed in these groups, as most of the defect was filled with newly formed bone tissue, being osteosynthesis active areas visible at the inner margins of the defect (Figure 2).

**Figure 4.**
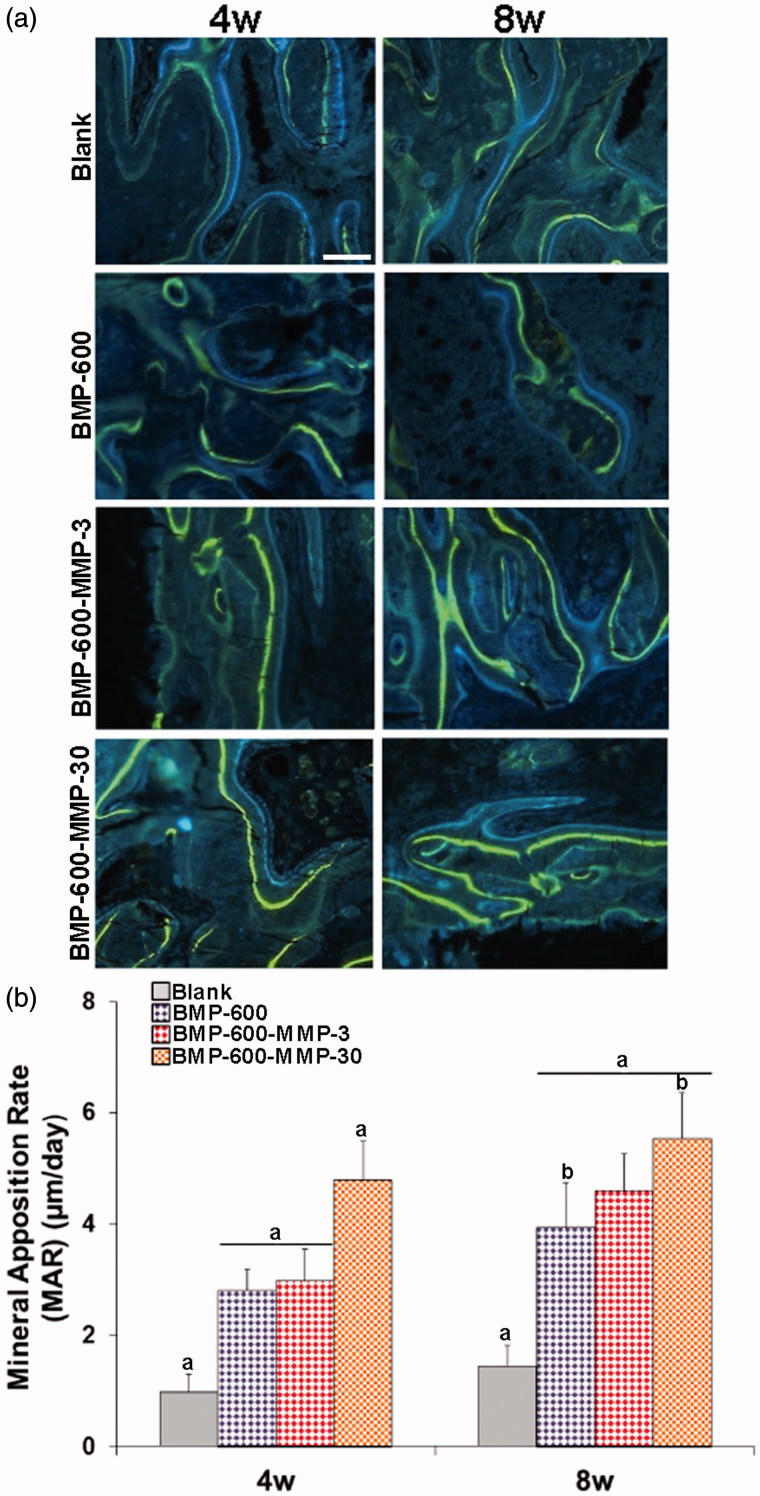
Mineral apposition rates determined in histological specimens from mouse calvaria at 4 and 8 weeks post-implantation in all the experimental groups. (a) Fluorochrome labeling (tetracycline and calcein) of the mineralization front. Doses were given 12 and 4 days prior to euthanasia and inter-label thickness was determined by image analysis to calculate the mineral apposition rate. Scale bar: 50 µm. (b) Quantification of mineral apposition rates within the ROI of all experimental groups. Data presented as means ± SD. The identical letter on different bars indicates significant differences (*n* = 4), *p* < .05.

Mineral apposition rate (MAR) was assessed in groups with highest repair, showing clear double labels of tetracycline (blue) and calcein (green), demonstrating new bone formation at 4 and 8 weeks post-surgery (Figure 4(a)). As expected, mineralization rate in the animals receiving empty microspheres was lower than in the groups treated with BMP-2 and the combination of BMP-2 and MMP10 (*p* < .05). Interestingly, MAR observed in BMP-600-MMP30 (ratio 20:1) group was higher (*p* < .05) than that achieved in the rest of the groups (Figure 4(b)). Eight weeks after implantation, a further increase in mineralization was observed but the differences in MAR between the treated groups were less pronounced than those found at 4 weeks (Figure 4(b)). Extensive fluorochrome labels were found throughout the specimen of the treated groups, whereas the scaffold alone and the empty defect only stimulated bone repair in the periphery of the defect (Figure 4(a)).

## Discussion

In the present study, we report for the first time that MMP10 enhances the reparative BMP-2 response *in vivo* in a murine model of critical-size calvarial bone defect, and increases the mineral apposition rate. MMP10 had been previously reported to interact with the BMP-2 signaling pathway in myoblastic cells *in vitro* (Mao et al., 2013). This evidence and our previous experience with BMP-2 in regeneration of critical bone defects (Rodríguez-Évora et al., 2013, 2014; Del Rosario et al., 2015a,b) led us to design and conduct the present study.

Sustained release of active substances from the artificial systems is critical to guarantee the permanence of the substances in the defect and, as a consequence, the maintenance of therapeutic efficacy might be expected. The injectable system proposed in our study, pluronic hydrogel, was able to sustain MMP10 and BMP-2 release pre-encapsulated in microspheres of PLGA. Maintenance of active substances in the systems is decisive to guarantee sustained therapeutic efficacy of the delivered substances avoiding the excessive burst release. Although a prolonged and sustained release of proteins was observed, a reduction of burst effect is still desirable. The fast release entails the loss of a certain fraction of the dose that is no longer available to exert its therapeutic action.

In this study, we showed first the effect of the dose of BMP-2 in the process of repairing a critical-size bone defect in a mouse model. A notable regenerative effect of the 300 and 600 ng of BMP-2 was observed after 4 weeks of treatment. However, the lowest dose of BMP-2 showed no better response than the control and blank groups. Moreover, the regenerative effect of 600 ng did not increase by adding 3 ng of MMP10 (200:1), but a significant increase in the repair rate was produced by adding 30 ng of MMP10 (20:1). MMP10 had been shown by other authors to enhance BMP-2-induced osteoblast differentiation *in vitro* (Mao et al., 2013), and our results confirm that a similar range of dose and proportions of BMP-2 and MMP10 is required *in vivo* to attain their synergistic osteogenic effect. After 8 weeks of treatment, the repair response in the BMP-600-MMP30 group remained the highest but no differences were found in the other treated groups except for BMP-100, B and C.

The role of BMP-2 as an inducer of osteogenesis and its effects promoting osteoblastic differentiation during bone repair processes are well known (Dimitriou et al., 2005; Devescovi et al., 2008; Kolambkar et al., 2011). In fact, BMP-2 is not only critical in bone formation but also in the process of vascular calcification, as it has been reported (Cheng et al., 2003; Li et al., 2008). However, in the present study, the combination of BMP-2 and MMP10 not only enhanced the repair response at shorter times but, even more importantly, increased significantly the mineral apposition rate throughout the experimental period. Being this a novel result, it was not completely unexpected since MMP10 had been previously reported to have a relevant role in epithelial wound healing (Gill & Parks, 2008) and its bone expression pattern suggested a potential role in endochondral ossification (Bord et al., 1998). Interestingly, MMP10 alone had been reported to have negligible effects on osteogenic marker expression, but its interaction with BMP-2 pathway increased the levels of Osterix, type 1 collagen, osteocalcin, and alkaline phosphatase (ALP) mRNA, as well as ALP activity in the myoblastic cell line C2C12 (Mao et al., 2013).

MMPs have been involved in bone metabolism and osteogenesis by either cleaving ECM components, shedding bioactive molecules from cell membranes or proteolytically modifying them (Aiken & Khokha, 2010). Since an efficient tissue fibrinolytic system has been shown to be essential for fracture repair (Yuasa et al., 2015), MMP10 ability for enhancing endogenous fibrinolysis (Orbe et al., 2011) could explain the enhanced reparative response. This would be in agreement with previous studies demonstrating the involvement of MMP10 in the tissue-repair process of the skeletal muscle and liver (Bobadilla et al., 2014; García-Irigoyen et al., 2014). Moreover, chemokines released from inflammatory cells are necessary to initiate bone repair (Bischoff et al., 2015) and several MMPs have been shown to process them, subsequently modifying their function (Gill & Parks, 2008). Since MMP3 (stromelysin-1, 82% homologous to MMP10) has been reported to cleave CCL2, CCL7, CCL8, CCL13, CXCL7, and CXCL12 (Gill & Parks, 2008), it would be conceivable that MMP10 could also take part by chemokine cleavage, however, there are no reports in this respect.

The main limitation of this study is that the mechanism by which MMP10 promotes the increase in mineralization is not known yet. It could be associated with an up-regulation of BMP-2 which, in turn, induces the expression of transcription factors responsible for osteoblastic differentiation and bone matrix production, such as Runx2 or Msx-2, as it has been demonstrated for other MMPs, such as MMP2 and MMP9 in the vascular smooth muscle calcification process (Shi et al., 2017). However, MMP10 has been shown to be required for proper repair in experimental models of tissue damage. CXCR4/stromal cell-derived factor-1 (SDF1)-regulated skeletal muscle repair has been shown to be dependent on MMP10 activity (Bobadilla et al., 2014), and crosstalk between MMP10 and the CXCR4/SDF1 axis has also been reported in a model of liver damage (García-Irigoyen et al., 2014). Interestingly, co-delivery of SDF1 enhances BMP-2-driven osteogenesis, associated with enhanced mobilization and homing of bone marrow-derived osteoprogenitor cells (Higashino et al., 2011; Sun et al., 2016). Thus, increased recruitment of osteoprogenitor cells through CXCR4/SDF1 signaling could be one of the mechanisms explaining MMP10-enhanced bone formation.

## Conclusions

The sustained release of the combination of MMP10 and BMP-2 in a ratio of 1:20 from the injectable and biodegradable delivery system made in this study enhanced bone healing and improved the mineralization rate.
